# Ultrasound evaluation of quadriceps muscle thickness and body fat distribution in children receiving maintenance hemodialysis: a cross-sectional case–control study

**DOI:** 10.1007/s00431-026-07212-x

**Published:** 2026-07-15

**Authors:** Amal E. Gohary, Azza Soliman Soliman, Sara Kamel Eldemerdash, Mona Mohammed Elsharkawy, Mahmoud M. Gohary

**Affiliations:** 1 Department of Pediatrics, Faculty of Medicine, Zagazig University, Zagazig City, Sharkia Governorate, Egypt; 2 Department of Public Health and Community Medicine, Faculty of Medicine, Zagazig University, Zagazig City, Sharkia Governorate, Egypt; 3 Department of Diagnostic Radiology, Faculty of Medicine, Zagazig University, Zagazig City, Sharkia Governorate, Egypt

**Keywords:** Fat distribution, Hemodialysis, Muscle ultrasound, Nutritional risk, Quadriceps muscle

## Abstract

Children on hemodialysis (HD) are at multifactorial nutritional risk. Nutritional assessment in these children is challenging because conventional anthropometric and dietary indicators are influenced by growth failure, chronic inflammation, and fluid shifts. Ultrasound (US)-based muscle assessment has emerged as a practical bedside method for evaluating muscle mass, while data on US-derived fat distribution in this population are limited. This cross-sectional case–control study was conducted at a tertiary HD center and enrolled 42 children on HD and 42 age- and sex-matched apparently healthy children. Demographic, anthropometric, clinical, and laboratory data were collected. Dietary evaluation using a 3-day food intake record was performed. Quadriceps muscle thickness, primarily that of the rectus femoris (RF), was assessed using B-mode muscle US in both groups. Subcutaneous thigh fat, anterior abdominal fat, and visceral fat thickness (VFT) were also assessed to characterize body composition. Total daily calorie intake and protein intake were significantly lower in children on HD (*p* < 0.001). Children on maintenance HD had significantly lower peripheral muscle thickness than healthy controls. Subcutaneous fat thickness at the thigh and abdominal wall was significantly reduced, whereas VFT was significantly increased in the HD group (*p* < 0.05). Correlation analysis showed that total caloric and protein intake were positively associated with muscle thickness (*p* < 0.001). Receiver operating characteristic (ROC) curve analysis showed that RF thickness demonstrated moderate discriminatory ability for nutritional risk, with an area under the curve (AUC) of 0.731 (*p* < 0.001), whereas US-derived fat measurements showed limited diagnostic performance.

*Conclusion*: Quadriceps muscle US appears to be a practical adjunct and complementary assessment tool to conventional nutritional assessment in children receiving maintenance HD. US-derived subcutaneous and visceral fat measurements provide complementary information on body composition and fat distribution.

**Table:** 

**What is Known:** • *Children receiving maintenance hemodialysis are at high risk of nutritional impairment, growth failure, and altered body composition, making nutritional assessment challenging.* • *Ultrasound assessment of quadriceps muscle thickness is a practical bedside method for evaluating muscle mass in adults receiving hemodialysis, but pediatric evidence remains limited.*	
**What is New:** • *This study highlights the utility of bedside US measurement of quadriceps muscle thickness as a practical adjunct for detecting nutritional risk in children on maintenance HD.* • *This study provides novel pediatric data on US-derived subcutaneous and VFT in children on maintenance HD, revealing an altered fat distribution pattern characterized by reduced peripheral fat and increased visceral fat.*

**Supplementary Information:**

The online version contains supplementary material available at 10.1007/s00431-026-07212-x.

## Introduction

Chronic kidney disease (CKD) is a significant global health issue. CKD is defined according to the degree of albuminuria and the glomerular filtration rate (GFR). Severe CKD requires renal replacement therapy (RRT), including dialysis or transplantation. RRT in children has been performed since the 1970s. About 50% of patients preparing for kidney transplantation require dialysis as a bridge. The proportion of patients receiving hemodialysis (HD) compared with peritoneal dialysis increases with age [1].

Assessment of nutritional status in children with CKD is challenging. It is important to remember that “children are not miniature adults.” Maintaining normal growth in children with CKD is an important goal. Assessment tools for nutritional status, such as dry weight, height/length, weight-for-height/length, body mass index (BMI)-for-height-age, and head circumference, are strongly recommended (grade A) for evaluating nutritional status in infants and children. Nutritional risk in children with end-stage kidney disease represents a multifactorial metabolic condition characterized by reduced protein reserves, impaired growth, inflammation, and altered body composition, and it cannot be explained solely by inadequate dietary intake [2–5].

The prevalence of nutritional problems in advanced CKD (stages IV–V) ranges from 50 to 75%, and poor growth has been associated with increased morbidity and mortality in children with CKD. A multidisciplinary team and a personalized approach are required for the management of nutritional risk in persons with CKD [6–8].

Perhaps the best definition of adequate nutritional status is the maintenance of normal body composition and a normal growth pattern through the consumption of appropriate types and amounts of food. Not all body composition measurements have reference data. In most studies in the current literature, a healthy reference group has been used [9, 10].

Diagnostic criteria for nutritional risk include low body weight, abnormal biochemical markers, inadequate caloric and protein intake, and reduced muscle mass. The 3-day food history is currently the gold standard for evaluating nutritional intake in children with CKD. However, it is not easy for respondents and caregivers, as it requires motivation, knowledgeable supervision, and assessment of usual intake over several days. Reduced muscle mass is one of the diagnostic criteria for nutritional problems according to newer definitions [11, 12].

Muscle mass can be assessed using various techniques to monitor and screen for muscle loss in high-risk populations. These methods, including bioimpedance analysis (BIA), enable the measurement of muscle mass but are affected by overhydration, a frequent problem in HD patients. The gold-standard techniques for assessing muscle mass include computed tomography (CT), dual-energy X-ray absorptiometry (DEXA), and magnetic resonance imaging (MRI). Currently, muscle ultrasound (US) has become a popular bedside technique for evaluating body composition, including muscle and fat assessment, because of its portability, affordability, accessibility, ease of use, and low training requirements [13–15]

According to recent research, quadriceps femoris US is a reliable and straightforward method for assessing muscle thickness in patients with kidney failure. Importantly, quadriceps measurements are relatively unaffected by rapid fluid shifts, making US a valuable tool for detecting muscle loss and assessing nutritional risk in HD patients [16, 17].

Research on the use of quadriceps US to support nutritional evaluation in children receiving HD is limited, and data on US-assessed body fat composition are also scarce. The objective of our study was to analyze the utility of US as an adjunct and screening method for evaluating nutritional risk and reduced muscle mass in children on maintenance HD and to assess body composition.

## Patients and methods

### Research design and patients

A matched cross-sectional study was conducted in the Pediatric Nephrology and HD Unit of a tertiary children’s hospital between October 2024 and October 2025. The study included 42 cases; all eligible patients were enrolled consecutively in the case group, and 42 matched healthy individuals were included as the control group. Children aged 1–16 years receiving regular maintenance HD for ≥ 6 months were eligible. Exclusion criteria included acute HD, known neuromuscular disorders, limb deformities affecting measurement, or refusal to participate.

Healthy controls were recruited from children attending routine pediatric outpatient visits or those admitted to the hospital for acute conditions. The control group was selected using individual age- and sex-matching. For each child receiving maintenance hemodialysis, a healthy control of the same sex and within ± 1 year of age was recruited whenever feasible. Inclusion criteria for the control group were age- and sex-matching with the patient group and the absence of chronic illness. Exclusion criteria included chronic systemic disease, obesity, known nutritional disorders, use of medications affecting body composition, and acute illnesses associated with dehydration, systemic inflammation, or reduced oral intake.

Sample size: a convenience sample was used due to the limited number of pediatric HD patients.

The sample size was determined as follows: the total number of dialysis cases referred to the pediatric HD unit was 42; therefore, all cases were included in the study as a consecutive sample for the case group, and 42 matched healthy individuals were included as the control group.

Ethics approval: following Institutional Review Board approval (ZU-IRB#751/20-Oct-2024), this study was approved by the Pediatrics Department at Zagazig University Hospitals. Informed consent was obtained from the legal guardians of all participants. This research was conducted in accordance with the ethical principles of the Declaration of Helsinki for research involving human participants.

### Biochemical measurements

Serum creatinine, blood urea nitrogen (BUN), uric acid (UA), cholesterol, total protein, albumin, calcium, and phosphorus (Pi) were measured using an automated chemical analyzer. Blood samples were collected immediately before the hemodialysis session (predialysis sampling) to minimize the effect of dialysis-related biochemical fluctuations. Biochemical analyses were performed in the central laboratory of the study institution using standardized automated laboratory methods [18]. These parameters were included to describe the clinical characteristics and were not used as diagnostic criteria for nutritional risk.

### Nutritional status assessment and definition of nutritional risk

Nutritional risk was defined using a composite assessment based on anthropometric indices and dietary intake according to Pediatric Renal Nutrition Taskforce (PRNT) recommendations; this composite definition was used as a proxy measure of nutritional risk rather than a formal diagnosis of protein-energy wasting, as full criteria application requires longitudinal data not available in this study.

Parents or patients were asked to complete a retrospective 3-day food intake recall. This involved recording everything the child drank and ate, including specific amounts, with a focus on daily energy and protein intake. The results were then compared with the recommended intake based on estimated energy requirements (EER) calculations, which accounted for sex, weight, age, and height, using the 50th percentile height-for-weight. Daily caloric and protein intake was compared with the Pediatric Renal Nutrition Taskforce (PRNT)–suggested dietary intake (SDI), which varies by age and sex [19]. Nutritional assessment and dietary adequacy were evaluated according to Pediatric Renal Nutrition Taskforce (PRNT) clinical practice recommendations [4, 7].

Anthropometric data were collected in accordance with WHO growth standards, along with dietary assessment. Calibrated scales were used to measure weight and height. To avoid edema-related weight bias, post-dialysis dry weight was estimated. Weight was measured using a sensitive platform scale, with the children wearing minimal clothing. Anthropometric measurements included weight, height, BMI, BMI-for-height-age, mid-upper arm circumference (MUAC), waist circumference, and skinfold thickness.

Given the high prevalence of short stature in children receiving HD, BMI interpretation was performed with consideration of height-age rather than chronological age when appropriate, using WHO growth standards. Nutritional risk was defined using conventional criteria, including anthropometric indices (BMI-for-height-age *z*-score <  − 2 SD and MUAC <  − 2 SD) and inadequate dietary intake based on PRNT recommendations [4]. US-derived muscle thickness was evaluated as an adjunct marker and was not used as a standalone diagnostic criterion.

### US measurements

A low-frequency curvilinear-array transducer (3.5 MHz) and a high-frequency linear-array transducer (7 MHz) were used with a Siemens ACUSON X300 ultrasound machine. All examinations were performed after completion of the hemodialysis session to minimize the influence of fluid overload and fluid shifts. Participants were positioned supine or slight lateral decubitus position as appropriate, and all measurements were performed on the right side.

### Muscle thickness measurements

Quadriceps muscle thickness was assessed for the rectus femoris (RF), vastus intermedius (VI), vastus medialis, and vastus lateralis muscles. Children were examined in the supine position with the leg semiextended and relaxed (Supplementary Fig. 1). A transverse (cross-sectional) approach was used at standardized anatomical landmarks. Muscle thickness was measured as the distance between the superficial and deep fasciae of each muscle at the midpoint and at the junction between the upper two-thirds and lower third of the distance from the anterior superior iliac spine to the upper pole of the patella [12].

### Fat thickness measurements

Fat distribution was evaluated by measuring visceral fat thickness (VFT), anterior abdominal subcutaneous fat thickness (AASFT), and subcutaneous thigh fat thickness (STFT). VFT was measured in the epigastric region using the 3.5-MHz transducer with the participant in the supine position and the knees slightly flexed. The distance from the peritoneal line to the posterior aspect of the rectus abdominis muscle was recorded. AASFT was measured using the 7-MHz transducer placed perpendicular to the skin in the midline above the umbilicus, and the hypoechoic layer between the skin surface and the anterior rectus sheath was recorded. STFT was measured at the mid-thigh level with the participant in the supine position and the leg extended, as the distance from the skin surface to the superficial fascia of the quadriceps muscle group.

### Data recording and analysis

All US measurements were performed by a single trained radiologist. All measurements were taken in triplicate, and the mean value was used for analysis. Care was taken to maintain a consistent probe pressure and angle to avoid measurement bias. The assessor was blinded to the clinical data to reduce measurement bias.

### Statistical analysis

Following data collection, Microsoft Excel 2016 for Windows, part of the Microsoft Office 2016 package (Microsoft Corporation, USA), was used to code the data into a spreadsheet. The data were then analyzed using IBM Statistical Package for the Social Sciences (SPSS) software, version 27 (IBM, USA). The normality of continuous data distribution was assessed using the Kolmogorov–Smirnov test. Data were presented as numbers and percentages for qualitative variables; means, standard deviations, and ranges for quantitative variables with a parametric distribution; and medians and interquartile ranges (IQRs) for quantitative variables with a nonparametric distribution.

For comparisons between two independent groups, Student’s *t*-test was used for normally distributed variables, and the Mann–Whitney *U* test was used for nonnormally distributed variables. The chi-square test was applied to assess associations between categorical variables. Correlation analysis was performed using Spearman’s correlation coefficient (*r*) to evaluate associations between quantitative variables. Receiver operating characteristic (ROC) curve analysis was used to determine the diagnostic performance of US measurements. A *p* value ≤ 0.05 was considered statistically significant [20]. The primary purpose of our exploratory ROC analysis was to evaluate whether ultrasound-derived muscle thickness could accurately identify children already classified as being at nutritional risk according to conventional clinical and dietary assessment methods.

Nutritional risk was defined using a composite reference standard based on anthropometric indices (BMI-for-height-age *z*-score <  − 2 SD and/or MUAC <  − 2 SD) and inadequate dietary intake, as defined by PRNT recommendations. The area under the curve (AUC), optimal cutoff values, sensitivity, specificity, positive predictive value (PPV), and negative predictive value (NPV) were calculated. A *p* value ≤ 0.05 was considered statistically significant.

A small proportion of data points was missing for laboratory and dietary variables: hemoglobin (2/42), albumin (1/42), and daily protein intake (3/42). Single imputation using mean substitution was applied because of the limited sample size. Missing values were assumed to be missing at random. Although multiple imputation is generally recommended, the very small proportion of missing data and the limited sample size reduced the anticipated benefit of more complex imputation approaches.

Ultrasound-derived muscle and fat thickness measurements were not included in the composite nutritional reference standard used to classify nutritional risk for ROC analysis.

## Results

### Enrollment and demographic characteristics

**Fig. 1:**
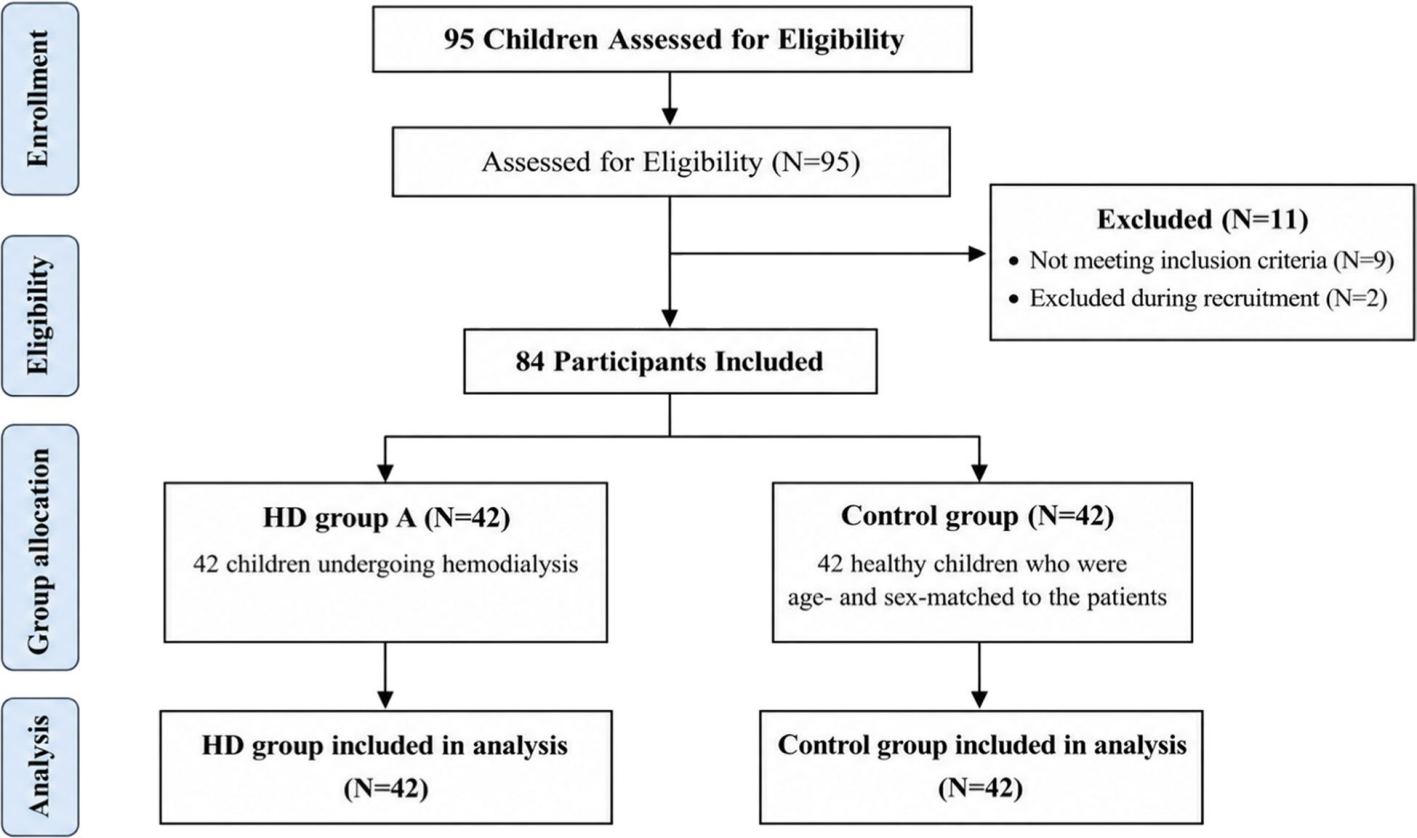
Flow diagram showing participant screening, eligibility assessment, inclusion, and allocation to the hemodialysis (HD) and control groups

This cross-sectional case–control study was conducted between October 2024 and October 2025. The participant selection process is illustrated in Fig. 1: a total of 95 children were assessed for eligibility. Eleven children were excluded, including 9 who did not meet the inclusion criteria and 2 who were excluded during recruitment. The remaining 84 participants were included in the study, comprising 42 children receiving maintenance hemodialysis (HD group) and 42 age- and sex-matched healthy controls. All included participants completed the study assessments and were included in the final analysis (Fig. 1).

### Patients’ baseline characteristics and nutritional evaluation

The study population included 42 children undergoing hemodialysis at the Pediatric Nephrology Unit of our tertiary care hospital children aged 1–16 years, representing a wide developmental range associated with physiological variation in muscle mass so 42 healthy children who were age- and sex-matched included as control group. The children undergoing maintenance hemodialysis had a mean age of 10.41 ± 3.29. Among hemodialysis cases, the most common underlying etiologies were glomerular diseases and CAKUT, tubulointerstitial disorders, and unknown etiology. The most frequent category was glomerular diseases and CAKUT (26.2%) followed by unknown etiology (23.8%). The median dialysis duration was 2 years (range 6 months–12 years) and median KT/V in cases 1.1.

Table 1 also demonstrates anthropometric assessment in hemodialysis patients; they had significantly lower weight SD for age (*p* = 0.009), length SD for age (*p* = 0.002), BMI SD for height age (*p* = 0.021), mid-arm circumferences (*p* < 0.001), compared to controls. However, no significant difference was observed in skin-fold thicknesses (*p* = 0.616) and waist circumference SDS (0.08). Future studies should consider the use of age-independent indices such as waist-to-height ratio.

**Table 1:** Demographic, anthropometric measurements and daily nutritional intake in HD children and control group

		HD cases (*N* = 42)		Controls (*N* = 42)		*p* value
		*N*	%	*N*	*%*	
Gender	Female	24	57.1%	22	52.4%	0.66^‡^
	Male	18	42.9%	20	47.6%	
Weight SDS for age	Median (IQR)	− 1.0 (− 2.0–0.0)		1.0 (− 0.5–2.0)		**0.009**^**╪**^
Length SDS for age	Median (IQR)	− 2.00 (− 3.0– − 1.0)		0.0 (− 1.0–0.0)		**0.002**^**#**^
BMI SDS for height age	Median (IQR)	− 1.0 (− 1.0–1.0)		1.0 (0.0–2.0)		**0.02**^**╪**^
MAC SDS (cm)	Median (IQR)	0.0 (− 1.0–1.0)		1.00 (1.0–2.0)		** < 0.001**^**╪**^
SFC SDS (mm)	Median (IQR)	1.00 (0.0–1.0)		0.59 (0.20–1.48)		0.61^#^
WC SDS (cm)	Median (IQR)	0.0 (− 1.0–1.0)		0.5 (0.0–0.5)		0.08^╪^
Total calories	Median (IQR)	1060 (859–1300)		1575 (1380–2000)		** < 0.001**^**╪**^
Protein intake (g/kg/day)	Mean ± SD	1.33 ± 0.64		1.73 ± 0.29		** < 0.001**^**#**^
Protein (%En)	Median (IQR)	12.1 (8.09–19.31)		17.2 (14.24–19.63)		**0.006**^**╪**^
Fat intake	Median (IQR)	1.6 (1.3–2.0)		1.8 (1.6–1.9)		0.12^╪^
Protein adequacy	Adequate	25	59.5%	95.0%		** < 0.001**^**‡**^
	Inadequate	17	40.5%	5.0%		
Caloric inadequacy	*N* (%)	7	(16.7%)	(0.0%)		**0.012**^**‡**^

*p* > 0.05 is nonsignificant; *p* ≤ 0.05 is significant. %EN: percentage of total daily energy intake derived from protein

*MAC* mid-arm circumference, *SFC* subcutaneous fat caliper, *WC* waist circumference, *SD* standard deviation score, *BMI* body mass index

^#^Student’s *t*-test

^╪^Mann–Whitney *U* test

^‡^Chi-square test

Values in bold indicate statistically significant results below (*p* 0.05)

A comparison of daily caloric and macronutrient intake between children on maintenance hemodialysis and healthy controls. The total daily calorie intake was significantly lower in HD children compared with the controls. Similarly, daily protein intake was significantly reduced in HD children (*p* < 0.001). In contrast, fat intake did not differ significantly between the groups (*p* = 0.120). The protein energy percentage was lower in HD cases, with a median of 12.1% versus 17.2% in controls (*p* = 0.006). Regarding protein adequacy, 59.5% of HD cases had adequate intake compared with 95% of controls (*p* < 0.001).

### Laboratory parameters of children and controls

Children on maintenance hemodialysis had end-stage kidney disease; eGFR was not calculated while eGFR in healthy controls was 95 (80–110) mL/min/1.73 m^2^.

As shown in Table 2, hemoglobin levels were significantly reduced in HD children (*p* = 0.001), indicating anemia, while total protein (*p* = 0.004) and albumin (*p* < 0.001) showed significant differences. Calcium and phosphorus concentrations were also significantly different, with HD children showing lower calcium levels (*p* = 0.002) and higher phosphorus levels (*p* < 0.001), indicating disturbances in mineral metabolism. Uric acid (UA) levels were elevated in the HD group (*p* < 0.001). Lipid profile analysis showed higher total cholesterol (149 mg/dL vs. 121 mg/dL, *p* < 0.001) and significantly lower HDL (56 mg/dL vs 76 mg/dL, *p* < 0.001) in HD group, while triglycerides (*p* = 0.310) and LDL (*p* = 0.805) did not differ significantly.

**Table 2:** Comparison between the two groups regarding laboratory parameters

	HD cases (*N* = 42)			Controls (*N* = 42)			Test value	*p* value^╪^
	Median	IQR		Median	IQR			
HB (g/dL)	10.2	9.2	11.0	11.0	10.9	11.2	3.45	**0.001**
Total protein (g/dL)	6.6	6.0	7.0	6.9	6.7	7.0	2.84	**0.004**
Albumin (g/dL)	3.7	3.6	3.8	4.2	4.0	4.5	6.16	** < 0.001**
Ca (mg/dL)	8.7	8.5	8.9	9.2	8.6	9.7	3.10	**0.002**
Pi (mg/dL)	5.6	5.0	6.8	4.5	4.1	4.8	5.16	** < 0.001**
Uric acid (mg/dL)	6.7	5.8	7.3	4.1	4.0	4.2	6	** < 0.001**
Cholesterol (mg/dL)	149.0	127.0	168.0	121.0	120.0	123.0	4.51	** < 0.001**
TG (mg/dL)	159.0	118.0	233.5	180.0	150.0	205.0	1.01	0.31
LDL (mg/dL)	35.0	28.0	45.0	36.0	35.0	37.0	0.24	0.80
HDL (mg/dL)	56.0	55.0	57.0	76.0	65.0	105.0	5.29	** < 0.001**

*p* > 0.05 is nonsignificant; *p* ≤ 0.05 is significant

^#^Student’s *t*-test

^╪^Mann–Whitney *U* test

Values in bold indicate statistically significant results below (*p* 0.05)

### Ultrasonographic assessment of muscle thickness and fat thickness among the studied groups

Table 3 demonstrates the comparison of US measurements of muscle thickness between children undergoing maintenance hemodialysis and healthy controls. Children receiving maintenance HD demonstrated significantly lower quadriceps muscle thickness across all assessed muscle groups compared with healthy controls including rectus femoris thickness (*p* = 0.026), vastus intermedius (*p* = 0.012), vastus medialis (*p* = 0.031), and vastus lateralis (*p* = 0.022). Subcutaneous fat (thigh and anterior abdominal) measurements also showed significant reductions in HD children, whereas visceral fat thickness was significantly higher among hemodialysis children (*p* < 0.001).

**Table 3:** Comparison of US assessment of muscle thickness and fat thickness among the two groups

		HD cases (*N* = 42)	Controls (*N* = 42)	Test value	*p* value
Rectus femoris thickness (mm)	Mean ± SD	8.81 ± 3.47	10.14 ± 3.24	2.22	**0.02**^#^
	Min–max	2.6–18.3	4.5–16.0		
Vastus intermedius (mm)	Median (IQR)	7.5 (5.7–10.6)	9.5 (7.0–13.0)	2.49	**0.01**^╪^
	Min–max	2.1–16.0	5.0–17.0		
Vastus medialis (mm)	Median (IQR)	12.0 (6.4–16.8)	15.0 (13.0–16.0)	2.16	**0.03**^╪^
	Min–max	6.4–31.0	8.0–22.8		
Vastus lateralis (mm)	Median (IQR)	14.95 (11.8–24.4)	20.0 (18.0–22.0)	2.29	**0.02**^╪^
	Min–max	9.8–39.2	11.0–27.0		
Thigh fat thickness (mm)	Median (IQR)	4.7 (2.6–5.5)	5.5 (4.5–7.0)	2.63	**0.008**^╪^
	Min–max	1.2–13.0	2.5–13.0		
Anterior abdominal fat thickness (mm)	Median (IQR)	6.70 (4.2–9.8)	8.0 (7.0–11.0)	2.01	**0.04**^╪^
	Min–max	2.4–36.5	3.8–36.7		
Visceral fat thickness (mm)	Median (IQR)	21.65 (17.1–25.9)	13.1 (12.0–16.0)	5.65	** < 0.001**^╪^
	Min–max	8.9–37.2	9.0–22.1		

*p* > 0.05 is nonsignificant; *p* ≤ 0.05 is significant

^#^Student’s *t*-test

^╪^Mann–Whitney *U* test

^‡^Chi-square test

Values in bold indicate statistically significant results below (*p* 0.05)

### Correlation of muscle with clinical, nutritional, and laboratory parameters in children on HD

As shown in Table 4, association analysis illustrated that total caloric intake was positively related to muscle thickness, including rectus femoris (*r* = 0.573, *p* < 0.001), vastus intermedius (*r* = 0.486, *p* = 0.001), and vastus medialis (*r* = 0.422, *p* = 0.006), but not significant with vastus lateralis (*r* = 0.290, *p* = 0.066). In addition, protein intake showed significant positive correlations with rectus femoris (*r* = 0.427, *p* = 0.005), vastus intermedius (*r* = 0.444, *p* = 0.004), vastus medialis (*r* = 0.514, *p* = 0.001), and vastus lateralis (*r* = 0.354, *p* = 0.023).

**Table 4:** Correlation of muscle and fat thickness with clinical, nutritional, and laboratory parameters in HD children

	Rectus femoris thickness (mm)		Vastus intermedius (mm)		Vastus medialis (mm)		Vastus lateralis (mm)		Thigh fat thickness (mm)		Anterior abdominal fat thickness (mm)		Visceral fat thickness (mm)	
	*r*	*p* value	*r*	*p* value	*r*	*p* value	*r*	*p* value	*r*	*p* value	*r*	*p* value	*r*	*p* value
Age (years)	0.04	0.76	− 0.01	0.94	0.06	0.69	0.04	0.76	0.33	**0.03**	0.22	0.16	0.12	0.43
Dialysis duration (years)	0.10	0.51	− 0.03	0.85	− 0.09	0.54	− 0.15	0.33	0.05	0.73	0.05	0.72	0.02	0.87
BMI	0.07	0.64	0.16	0.29	0.05	0.74	0.15	0.31	− 0.02	0.90	− 0.04	0.77	0.21	0.17
KT/V	0.09	0.53	− 0.18	0.23	− 0.07	0.66	− 0.12	0.42	− 0.03	0.84	0.09	0.55	− 0.07	0.62
Total calories	0.57	** < 0.001**	0.48	**0.001**	0.42	**0.006**	0.29	0.06	0.50	**0.001**	0.19	0.23	0.42	**0.006**
Protein intake	0.42	**0.005**	0.44	**0.004**	0.51	**0.001**	0.35	**0.02**	− 0.27	0.08	− 0.46	**0.002**	− 0.32	**0.03**
Fat intake	− 0.20	0.19	− 0.19	0.23	− 0.09	0.54	− 0.18	0.24	− 0.17	0.28	− 0.30	0.051	− 0.09	0.57
HB (g/dL)	0.07	0.66	− 0.14	0.36	− 0.02	0.88	− 0.15	0.32	0.33	**0.03**	0.24	0.11	0.09	0.57
Creatinine (mg/dL)	0.60	** < 0.001**	0.48	**0.001**	0.52	** < 0.001**	0.46	**0.002**	0.39	**0.009**	0.26	0.09	0.30	0.05
BUN (mg/dL)	0.008	0.96	0.26	0.09	0.13	0.41	0.19	0.21	0.11	0.46	0.007	0.96	0.14	0.35

*p* > 0.05 is nonsignificant, *r*: Spearman correlation coefficient

Values in bold indicate statistically significant results below (*p* 0.05)

Renal function markers, particularly creatinine, were positively correlated with all muscle thickness measurements: rectus femoris (*r* = 0.604, *p* < 0.001), vastus intermedius (*r* = 0.480, p = 0.001), vastus medialis (*r* = 0.526, *p* < 0.001), and vastus lateralis (*r* = 0.467, *p* = 0.002). Other laboratory parameters, including total protein, albumin, and lipid profile, showed no significant correlations with muscle thickness, as well as with thigh fat. Hemoglobin was positively correlated with thigh fat (*r* = 0.334, *p* = 0.031).

### *VROC analysis of ultrasound-derived muscle and fat thickness for identification of nutritional risk (*Table 5*)*

**Table 5:** Validity (AUC, sensitivity, specificity) of muscle and fat thickness in detection of malnutrition

	**Best cutoff** ^*^	**Sensitivity**	**Specificity**	**PPV**	**NPV**	**AUC**	**95%CI**	***p*** ** value**
Rectus femoris thickness (mm)	10.4	100%	48%	18.8%	100%	0.73	0.51–0.95	** < 0.001**
Vastus intermedius (mm)	8.3	88.9%	61.3%	21.6%	97.9%	0.70	0.48–0.93	**0.01**
Vastus medialis (mm)	14.4	77.8%	61.3%	19.4%	95.8%	0.67	0.44–0.90	**0.03**
Vastus lateralis (mm)	20.6	100%	52%	20%	100%	0.73	0.51–0.95	**0.001**
Thigh fat thickness (mm)	4.4	55.6	77.3%	22.7%	93.5%	0.57	0.34–0.80	0.50
Anterior abdominal fat thickness (mm)	7.6	66.7	54.7%	16%	91.5%	0.61	0.38–0.84	0.24
Visceral fat thickness (mm)	19	66.7	66.7%	19.4%	94.3%	0.57	0.34–0.80	0.48

*AUC* area under a curve, *NPV* negative predictive value, *PPV* positive predictive value

^*^Cutoff values were determined using the Youden index to maximize combined sensitivity and specificity. These thresholds are exploratory and were derived and evaluated within the same study cohort

Values in bold indicate statistically significant results below (*p* 0.05)

Exploratory ROC analysis was performed to evaluate the discriminatory ability of ultrasound-derived muscle measurements for identifying children at nutritional risk. In Table 5, among muscle parameters, rectus femoris thickness ≤ 10.4 mm showed high sensitivity within this cohort (100%); however, the overall discriminatory ability was moderate, as reflected by an AUC of 0.731 (*p* < 0.001); vastus lateralis thickness ≤ 20.6 mm showed moderate discrimination (AUC = 0.736, *p* = 0.001), with high sensitivity (100%) and moderate specificity (52%). Vastus intermedius ≤ 8.3 mm and vastus medialis ≤ 14.4 mm demonstrated mild discriminatory performance, with AUC values of 0.705 (*p* = 0.012) and 0.678 (*p* = 0.032), respectively (Table 5).

Subcutaneous and visceral fat measurements showed limited discriminatory performance for detecting nutritional risk. Thigh fat ≤ 4.4 mm had moderate specificity (77.3%) but low sensitivity (55.6%) and poor discrimination (AUC = 0.573, *p* = 0.501). Anterior abdominal fat ≤ 7.6 mm and visceral fat ≤ 19 mm showed limited sensitivity and specificity (66.7%/54.7% and 66.7%/66.7%, respectively) with nonsignificant AUC values (*p* = 0.240 and 0.481).

Among ultrasound-derived muscle parameters, rectus femoris and vastus lateralis thickness demonstrated moderate exploratory discriminatory performance for identifying nutritional risk, whereas fat thickness measurements showed limited discriminatory ability.

The positive correlation between serum creatinine and muscle thickness should be interpreted cautiously, as creatinine levels in hemodialysis patients are influenced by multiple factors beyond muscle mass.

## Discussion

Nutritional impairment, referred to in this study as nutritional risk, represents a major clinical challenge that becomes increasingly prevalent with advancing stages of chronic kidney disease (CKD), with a high occurrence in patients on maintenance dialysis, increasing the risks of morbidity, mortality, and healthcare costs [21].

Nutritional US is a useful tool for evaluating both muscle mass and muscle quality. US assessment of quadriceps femoris muscle thickness is highly reliable in HD patients and can accurately reflect nutritional status [22]. To our knowledge, this topic has not previously been studied in the pediatric age group. In this study, 42 children on regular HD and 42 healthy controls were included (Fig. 1). According to the clinical evaluation, children on HD were found to have significantly lower weight, height, mid-arm circumference, and skin-fold thickness than controls (*p* < 0.001). This finding is consistent with earlier research showing that growth is significantly impaired and that the severity of growth impairment increases with the duration of HD [23].

In our study, 38% of cases had under built (weight below − 2 SD for age), and 59.5% had short stature (height below − 2 SD for age), while only 7.1% of participants had a BMI below − 2 SD for height age, as height is more affected than weight (Supplementary Table 1). This agrees with many studies reporting growth failure in late-stage renal failure, where treatment of comorbidities, improved diet, and metabolic control are important components of management [24, 25].

In our study, children on maintenance hemodialysis had significantly lower weight, height, BMI, mid-arm circumference, skin-fold thickness, and subcutaneous fat thickness than healthy controls (*p* < 0.05), whereas waist circumference did not differ significantly. In contrast, visceral fat thickness was significantly higher in the HD group (*p* < 0.001), suggesting preferential central fat accumulation. However, these findings should be interpreted cautiously because controls were recruited from outpatient clinics and hospital admissions for acute conditions, which may have influenced body composition [26].

Several studies examining body composition in adult hemodialysis populations have described the concept of sarcopenic obesity, characterized by reduced muscle mass accompanied by increased visceral fat. This altered body composition pattern has been linked to accelerated atherosclerosis and higher morbidity and mortality in individuals undergoing hemodialysis. Evidence shows that HD patients tend to accumulate abdominal visceral fat while simultaneously losing lean body mass, regardless of their body mass index; these findings are consistent with the results of our study [27].

US methods offer the advantage of easy bedside application for assessing skeletal muscle, with the quadriceps being the largest muscle group and therefore ideal for evaluating muscle mass. In a previous study of 121 patients with ESRD receiving HD, patients exhibited lower QVIT and QRFT than both control groups (*p* < 0.001). That study showed that skeletal muscle US is a simple bedside method that can be used in HD units and is a helpful tool for assessing muscle mass [12, 28].

In our study, children on HD were compared with controls using US measurements of quadriceps muscle thickness. RF thickness ≤ 10.4 mm showed moderate diagnostic performance for identifying nutritional risk (AUC = 0.731, *p* < 0.001), with high sensitivity (100%) but limited specificity (48%) (Table 5) (Supplementary Fig. 2). In addition to muscle assessment, US evaluation of subcutaneous and visceral fat provided complementary information on body fat distribution. Children on HD showed reduced peripheral subcutaneous fat thickness but increased VFT compared with healthy peers, suggesting an altered fat distribution pattern with relative central adiposity. However, in contrast to quadriceps muscle thickness, fat US parameters demonstrated limited diagnostic performance for identifying nutritional risk, with low sensitivity and nonsignificant AUC values. These findings indicate that US-derived fat measurements should be considered descriptive markers of body composition rather than screening tools for nutritional risk in this population [14, 16, 26, 29].

Several limitations should be considered when interpreting these findings. Although age- and sex-matched controls were included, age-specific ultrasound reference values were unavailable across the full pediatric age range, pubertal status was not assessed, and multivariable adjustment was not performed because of the relatively small sample size. Furthermore, ultrasound-derived muscle and fat thickness measurements were not included in the composite nutritional reference standard used to classify nutritional risk for ROC analysis. Because the ROC-derived thresholds were developed and tested within the same cohort, overfitting cannot be excluded. The reported cutoffs should be externally validated in larger, multicenter pediatric hemodialysis cohorts before these thresholds can be considered for clinical application.

In our HD cohort, 38% of children had weight below − 2 SD for age, 59.5% had height below − 2 SD for age, and only 7.1% had BMI below − 2 SD for height-age. Caloric inadequacy was identified in 16.7% of patients using chronological age and 42% using height-age, while 40% had inadequate protein intake. In contrast, ultrasound-derived muscle thickness identified low muscle mass in approximately 50% of patients (Supplementary Table 1). These findings suggest that muscle ultrasound may identify a greater proportion of children at nutritional risk than conventional anthropometric and dietary assessments, whereas BMI appears relatively insensitive in this population because CKD disproportionately affects linear growth compared with body weight [28, 30].

In our study, total caloric and protein intake was positively associated with muscle thickness. Although serum creatinine was positively correlated with all muscle thickness measurements, this association should be interpreted cautiously because creatinine levels in children receiving maintenance hemodialysis are influenced by dialysis adequacy, residual renal function, and metabolic factors in addition to muscle mass. Similar findings have been reported in adults undergoing maintenance hemodialysis [31]. Furthermore, quadriceps muscle thickness appears relatively unaffected by acute fluid shifts, as a meta-analysis of 15 studies involving 1584 hemodialysis patients demonstrated significantly lower muscle thickness in HD patients than in healthy controls, with no significant differences before and after dialysis sessions [16, 17].

Differences in nutritional risk prevalence across assessment methods likely reflect their differing sensitivities and target domains. While anthropometric and dietary assessments primarily identify established nutritional impairment, muscle ultrasound may detect earlier changes in muscle mass. Therefore, muscle ultrasound should be considered a complementary tool to conventional nutritional assessment, particularly in children receiving hemodialysis with altered body composition and fluid shifts.

## Strengths, limitations, and recommendations

This study has several notable strengths. It addresses nutritional risk and the early detection of nutritional impairment in children receiving hemodialysis, a population for which comparative data remain limited. In addition, it evaluates the role of ultrasound as a practical bedside tool that is readily available in most HD units and may facilitate the assessment of muscle mass and the early identification of children at nutritional risk.

However, several limitations should be considered. The single-center design, relatively small sample size, and cross-sectional nature of the study limit the generalizability of the findings and preclude assessment of causality implications. Furthermore, comparison with gold-standard methods for muscle mass assessment was not feasible. Due to financial constraints, concerns regarding unnecessary radiation exposure, and the absence of longitudinal follow-up, computed tomography (CT)–based muscle assessment could not be performed for validation purposes.

The wide age range of participants also represents a limitation, as pubertal status was not assessed and may have influenced muscle thickness measurements. Nevertheless, the use of age- and sex-matched controls, together with standard deviation scores (SDS) for anthropometric parameters, likely minimized the impact of age-related variability on the comparative results.

The selection of the control group may have introduced bias, as healthy controls were recruited from outpatient visits and hospital admissions for acute conditions. Such conditions could have influenced hydration status, dietary intake, or body composition; thus, the control participants may not be fully representative of the general healthy pediatric population.

Although all ultrasound examinations were performed by a single experienced radiologist using a standardized acquisition protocol, formal assessment of intraobserver reliability, interobserver reliability, and coefficients of variation was not performed. Consequently, measurement reproducibility could not be quantified and should be evaluated in future multicenter studies.

The use of single imputation for missing data may have slightly reduced statistical precision; however, given the small proportion of missing values, this is unlikely to have significantly affected the overall findings. Sensitivity analyses excluding imputed data yielded comparable results.

Reduced quadriceps muscle thickness in children receiving HD likely reflects a combination of diminished protein stores and a chronic catabolic state, rather than physical deconditioning alone. Nevertheless, physical activity was not objectively assessed in this study. Reduced activity levels and deconditioning are common in children on maintenance HD and may independently contribute to decreased muscle mass. Therefore, residual confounding related to physical activity cannot be excluded. Future studies should incorporate standardized pediatric physical activity assessments or wearable monitoring devices to better delineate the relative contributions of inactivity and nutritional risk to muscle loss.

The relatively small number of children classified as having nutritional risk may have reduced the precision of ROC-derived cutoff estimates and contributed to wide confidence intervals and the proposed ultrasound cutoff values were derived from the present cohort and have not undergone external validation; therefore, they should be interpreted as exploratory findings. The possibility of overfitting cannot be excluded because threshold derivation and performance evaluation were performed in the same cohort so these findings require validation in larger multicenter cohorts.

The composite nutritional reference standard used in this study does not fully capture the multidimensional nature of nutritional risk in pediatric CKD. Misclassification of nutritional status may therefore have occurred and may have influenced the observed ROC performance.

Future research should focus on establishing age-specific normative reference values for ultrasound-based muscle assessment and on evaluating the relationship between ultrasound-derived muscle parameters, functional outcomes, and long-term clinical prognosis in children with CKD. In addition, the proposed ultrasound cutoff values should be externally validated in larger, independent, multicenter pediatric hemodialysis cohorts before consideration for routine clinical use. This is particularly relevant given the occurrence of HD-related complications, including two deaths observed during the study period.

Despite these limitations, the present findings support the feasibility and potential clinical utility of ultrasound-based muscle assessment as part of a comprehensive, multidimensional approach to nutritional evaluation in pediatric dialysis patients.

## Conclusion

Ultrasound assessment of quadriceps muscle thickness appears to be a practical adjunct and should be considered a complementary assessment tool alongside conventional nutritional evaluation in children receiving maintenance hemodialysis.

Ultrasound-derived measurements of subcutaneous and visceral fat provide complementary insights into body composition and fat distribution; however, they are not suitable as standalone screening tools for nutritional risk.

Further longitudinal studies are warranted to clarify the role of ultrasound in diagnosis and to determine its utility in guiding nutritional interventions.

## Supplementary Information

Below is the link to the electronic supplementary material.

ESM 1 (DOCX 743 KB)

## Data Availability

No datasets were generated or analysed during the current study.
