# Supplementary material for: Ultrasound evaluation of quadriceps muscle thickness and body fat distribution in children receiving maintenance hemodialysis: a cross-sectional case–control study
Source: Eur J Pediatr. 2026 Jul 15;185(8):577. doi: 10.1007/s00431-026-07212-x (PMC13373012; doi:10.1007/s00431-026-07212-x)
Supplement: Supplementary file 1 — (DOCX 743 KB) [file 431_2026_7212_MOESM1_ESM.docx]

**Supplementary materials**

**Supplementary Table 1: Prevalence of malnutrition according to different assessment modalities in HD group:**

As summarized in Supplementary Table 1:in cases group 38% of children had a weight-for-age below −2 SD, and 59.5% had a height-for‑age below −2 SD, while only 7.1% had a BMI-for–height-age below −2 SD. Caloric inadequacy was detected in 16.7% of patients when evaluated by chronological age but increased markedly to 42.9% when adjusted for height age. Additionally, 40.5% of patients demonstrated insufficient protein intake. In contrast, using U.S.-derived muscle ultrasound cutoff values, low muscle mass was detected in approximately 50% of patients. These findings suggest that muscle ultrasound may identify a higher proportion of children at risk of malnutrition compared with conventional anthropometric or dietary assessments.

**Supplementary Table 1:Prevalence of malnutrition according to different assessment modalities in children on maintenance HD in comparison to muscle ultrasound cut off (n = 42) :**

| **Parameter** | **Definition** | **n (%)** |
| --- | --- | --- |
| Weight SD for age | Weight z-score < −2 SD | 16 (38) |
| Length SD for-age | Length z-score < −2 SD | 25(59.5) |
| BMI SD for-age height | BMI z-score < −2 SD | 3 (7.1) |
| Caloric intake (chronological age) | Intake < estimated energy requirement (EER) | 7 (16.7) |
| Caloric intake (corrected age for height) | Intake < height-age–adjusted EER | 18 (42.9) |
| Protein intake inadequacy | Intake below age-specific recommendations | 17 (40.5) |
| Low muscle mass (ultrasound-based) | Below predefined quadriceps muscle thickness cut-off | 21 (50.0) |

Data are presented as number (percentage). EER, estimated energy requirement. Muscle mass was assessed using quadriceps muscle ultrasonography

**Supplementary Figure 1:Muscle Ultrasound Technique And Findings**

**[A] Probe Position During Quadriceps Muscle Ultrasound Assessment.**

Supplementary Figure 1 demonstrates the correct placement of the linear ultrasound transducer over the anterior thigh for quadriceps muscle assessment. The probe is positioned perpendicular to the skin surface at the midpoint of the thigh while the operator stabilizes the transducer with a gloved hand to ensure consistent contact and minimize compression of the underlying muscle tissue. This standardized positioning is essential for obtaining accurate and reproducible measurements of quadriceps muscle thickness.


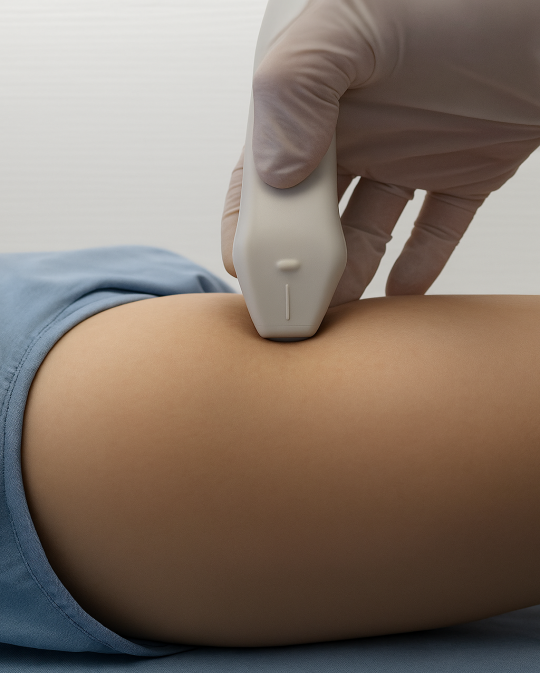


**[B]Ultrasound image of right thigh muscle thickness measurement in HD patient**

Transverse ultrasound image of right mid thigh in hemodialysis patient shows 1.sub cutaneous fat thickness 2.Rectus femoris thickness 3.Vastus intermedius thickness 4.Vastus lateralis thickness


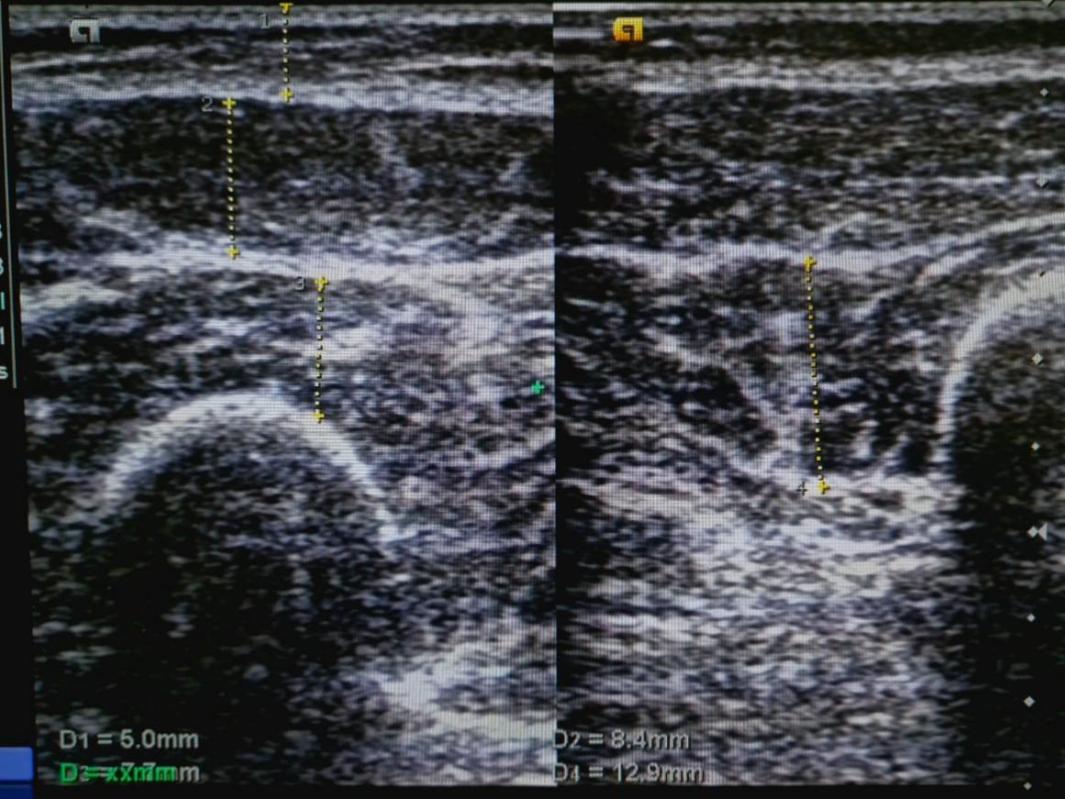


**[C] Right thigh quadriceps muscle thickness and fat thickness in control patient**


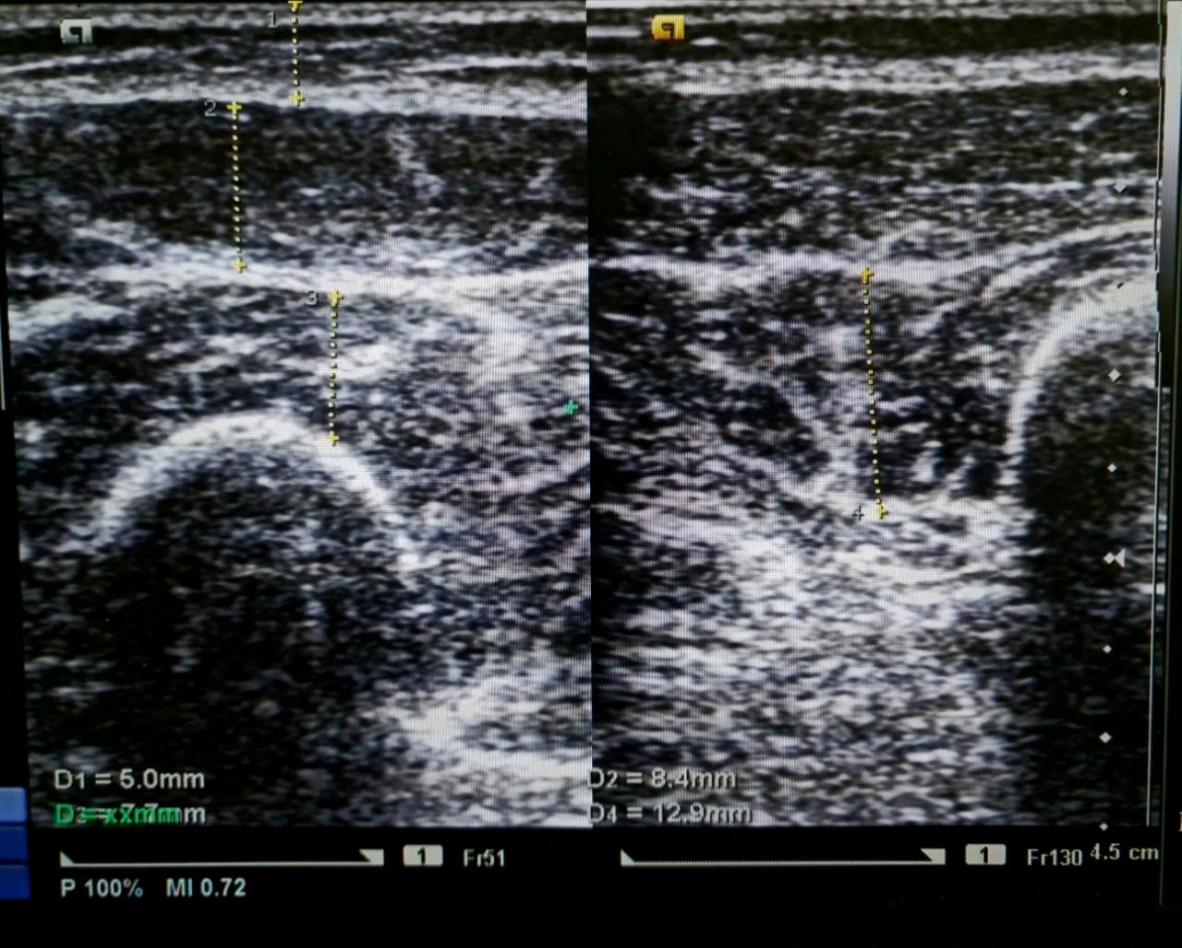
Transverse ultrasound image of right mid thigh in control subject shows 1.sub cutaneous fat thickness 2.Rectus femoris thickness 3.Vastus intermedius thickness 4.Vastus lateralis thickness

**Supplementary Figure 2: (Receiver Operating Characteristic - ROC Curve) of ultrasound-derived quadriceps muscle thickness for identification of nutritional risk in children receiving maintenance hemodialysis.**

Receiver operating characteristic (ROC) analysis was performed to evaluate the ability of ultrasound measurements to detect nutritional risk in children on maintenance hemodialysis (Figure1). Among muscle parameters, Rectus femoris thickness ≤10.4 mm demonstrated moderate discriminatory ability as reflected by an AUC of 0.731 (p < 0.001)


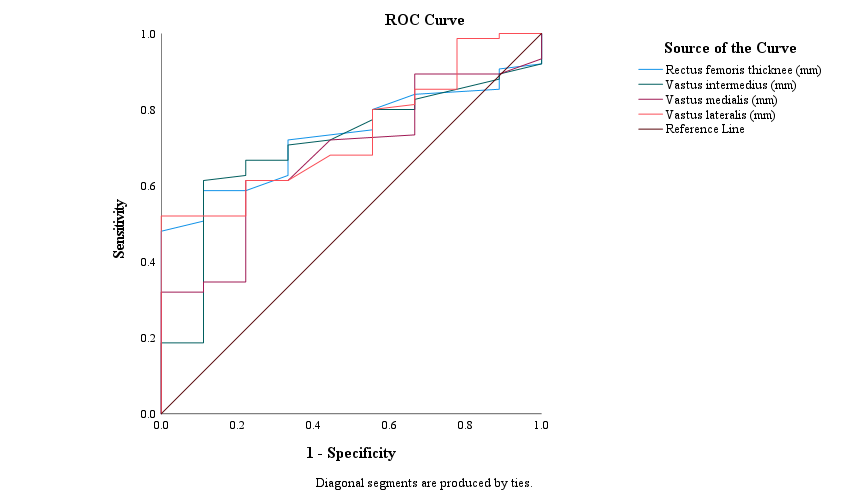


**Supplementary Figure (2): showed ROC curve of muscle thickness for identification of nutritional risk**
